# Adjuvanted Influenza Vaccines Elicits Higher Antibody Responses against the A(H3N2) Subtype than Non-Adjuvanted Vaccines

**DOI:** 10.3390/vaccines8040704

**Published:** 2020-11-25

**Authors:** Laura Sánchez de Prada, Iván Sanz Muñoz, Javier Castrodeza Sanz, Raúl Ortiz de Lejarazu Leonardo, José María Eiros Bouza

**Affiliations:** 1 Hospital Clínico Universitario de Valladolid, 47003 Valladolid, Spain; jjcastrodeza@saludcastillayleon.es; 2 Centro Nacional de Gripe de Valladolid, 47009 Valladolid, Spain; isanzm@saludcastillayleon.es (I.S.M.); lejarazu@gmail.com (R.O.d.L.L.); jmeirosbouza@gmail.com (J.M.E.B.); 3 Hospital Universitario Río Hortega de Valladolid, 47012 Valladolid, Spain

**Keywords:** influenza A, Vaccines, adjuvanted Vaccines

## Abstract

Background: vaccination is the best approach to prevent influenza infections so far. Serological studies on the effect of different vaccine types are important to address vaccination campaigns and protect our population. In our study, we compared the serological response against influenza A subtypes using the non-adjuvanted influenza vaccine (NAIV) in adults and the elderly and the adjuvanted influenza vaccine (AIV) in the elderly. Methods: We performed a retrospective analysis by hemagglutination inhibition assay (HI) of serum samples right before and 28 days after seasonal influenza vaccination during the 1996–2017 seasons. Conclusions: The AIV presents better performance against the A(H3N2) subtype in the elderly whereas the NAIV induces a better response against A(H1N1)pdm09 in the same group.

## 1. Introduction

Influenza viruses circulate widely, causing seasonal epidemics in both hemispheres during the winter months. This disease causes 2–5 million hospitalizations and over 650,000 deaths worldwide each year [1].

The presentation of influenza disease ranges from an asymptomatic infection to a fulminant illness, depending on the characteristics of both the host and virus. The incubation period lasts 1–2 days after which, suddenly, unspecific systemic symptoms are developed. Fever, chills, headache, myalgia, malaise and anorexia are part of the clinical presentation that usually comes along with respiratory symptoms, including a non-productive cough, nasal discharge and sore throat [2].

Most people recover from uncomplicated influenza, but complications can result in severe illness and death, particularly among children younger than 2 years; older adults; pregnant and postpartum women within 2 weeks of delivery; people with chronic underlying conditions, such as pulmonary, cardiac and metabolic diseases; as well as immunocompromised patients [3].

To date, the annual influenza vaccination is the best method to limit the transmission and severity of infection. It is estimated that vaccination of risk groups could decrease the in-hospital deaths by 52–79% and cause a 37% reduction in ICU admissions [4]. Influenza Vaccines are strain-specific, and the immunity induced is inversely correlated with the antigenic distance between the circulating and vaccine strains [5]. The continuous antigenic drift of influenza viruses obliges one to revise the Vaccines’ composition each year, update the strains frequently and vaccinate the population each year. 

At the present time, there are different influenza Vaccines being used across the world, which differ in the way of manufacturing (eggs or cell culture), the number of influenza viruses included (trivalent and quadrivalent) and also based on their capacity to enhance the immune response on the population vaccinated (adjuvanted and non-adjuvanted), among others. Adjuvanted influenza Vaccines (AIV) are designed to enhance immune responses, and usually they are employed in certain risk groups that need higher protection, like the elderly [6,7]. The adjuvants contained in this kind of vaccine increase the immune response. One of the most experienced and safe adjuvants known is MF59, an oil-in-water emulsion, inviscid and easy to inject. This adjuvant induces cytokine secretion and cell recruitment in the site of injection, which leads to further antigen capture [8]. The adjuvanted vaccine induces a long-term immune response by the memory cells, highly protective antibody titers and a higher degree of cross-reactive immunization [9]. 

In the elderly, immune-senescence processes combined with chronic conditions make them one of the main targets for seasonal influenza vaccination. Although this population group does not account for the highest prevalence of influenza disease during seasonal epidemics, they are the age group with the highest hospitalization and mortality rates. That is the reason that makes the elderly a group of special interest in order to identify the particular requirements for increasing the efficacy of the vaccination [7].

The aim of this study is to compare the humoral response against influenza A viruses of the adjuvanted and the non-adjuvanted influenza vaccine in the elderly and compare them with a group of adults vaccinated with the non-adjuvanted vaccine.

## 2. Materials and Methods

### 2.1. Patient Recruitment

A retrospective observational study was designed and a total of 3585 healthy individuals was recruited from the vaccination programs conducted by the Influenza Sentinel Surveillance Network of Castile and Leon (Spain) (ISSNCyL) during the Influenza vaccine Campaigns (IVCs) between 1996 and 2017; therefore, some of these people probably may have been vaccinated repeatedly some years, consecutively or not. Their health status was documented by clinicians during medical visits. This population was divided in three study groups: patients of 15–64 years old who received a non-adjuvanted influenza vaccine (NAIV; *n*_1_ = 813); patients ≥65 years who received an NAIV (*n*_2_ = 1561); and patients ≥65 years who received an adjuvanted influenza vaccine (AIV; *n*_3_ = 1211). Patients of 15–64 years old were included as an age control for comparison to the elderly groups. The selection of these age-cohort criteria was carried out based on the habitual age groups used in influenza studies. Serum samples were obtained by the clinicians of the ISSNCyL and stored at −20 °C before being sent for analysis to the National Influenza Centre of Valladolid (Spain). Pre-vaccination sera were obtained right before influenza vaccination and post-vaccination samples at a minimum of 28 days after vaccination to ensure a correct immunization. The delivered seasonal influenza Vaccines included the A and B influenza strains recommended by the World Health Organization (WHO) for the Northern Hemisphere for each IVC. All subjects gave their informed consent for inclusion before they participated in the study, and the recruitment of patients was performed following the Spanish Organic Law for Data Protection, patient´s rights and obligations for clinical documents (BOE n°298 of 14 December 1999, Law 41/2002). This research was performed according to the Declaration of Helsinki, and was yearly approved by the Community Ministry of Health of Castile and Leon (Spain).

### 2.2. Hemagglutination Inhibition Assay (HI)

The presence of anti-hemagglutinin antibodies (Abs) in pre- and post-vaccination serum samples was analyzed by performing the hemagglutination inhibition assay (HI). This analysis was conducted following the protocol recommended by the WHO and the Influenza Surveillance Network for the surveillance of influenza viruses and vaccine efficacy [10]. Beforehand to the HI, the serum samples were treated combining 100 µL of serum with 300 µL of RDE (Receptor Destroying Enzyme; Denka Seiken, Tokyo, Japan) to remove non-specific inhibitors from the sera. This RDE–serum combination was incubated at 37 °C in a water bath for 18 h and then inactivated at 56 °C for 1 h. Then, this combination was diluted in PBS to a work concentration of 1/10. To perform HI, serial double dilutions of 50 µL of each serum were conducted in 96-V-microwell plates, and then 50 µL of the virus standardized to 4 haemagglutinin units (4HU) was incorporated into each well and incubated for 30 min at room temperature. Finally, 50 µL of hen erythrocytes at 0.75% were added and incubated at room temperature for another 30 min. The Abs titer was defined as the highest dilution causing complete hemagglutination inhibition. Pre- and post-vaccination titers were included in the database for their study. For this analysis, the classical A(H1N1), A(H1N1) pdm09 and A(H3N2) vaccine strains designed by the WHO for each IVC were used. A PBS negative control and a viral-only positive control were used in each plate. A serum control was also included for each sample, which included only the serum sample without a virus to assess the presence of unspecific inhibitors.

### 2.3. Statistical Analysis

The results were analyzed by using the classical serological criteria of the European Medicament Agency (EMA) for the evaluation of vaccine efficacy [11]. Those criteria evaluate different parameters, such as the seroprotection rate (SPR) (percentage of individuals with antibody titers ≥ 1/40), seroconversion rate (SCR) (percentage of individuals showing at least a four-fold induction of pre-vaccination titers) and the geometric mean titers (GMTs) and their increase. This increase was calculated dividing the post-vaccine GMTs and pre-vaccine GMTs. Negative results in HI were assumed as half of the detection threshold (1/10). Even though some studies suggest that higher protective titers must be applied to a population ≥65 years old when evaluating seroprotection, the current global consensus was followed, and a titer of 1/40 was considered as protective [12,13]. Seroconversion was defined as a titer increase of at least four-fold between pre- and post-vaccination sera. In addition, seroconversion was considered to have occurred in cases of negative pre-vaccination sera that achieved 1/40 titers after vaccination. Different statistical non-parametric tests were used, using SPSSV20 (IBM, Armonk, NY, USA), and taking statistical significance at the *p* < 0.05 value.

## 3. Results

### 3.1. Population Characteristics

The median age of each group recruited for this study was 55 years old (SD: 11.0) for patients of 15–64 years old who received an NAIV, 75 (SD: 7.9) in patients ≥65 years who received an NAIV and 81 (SD: 8.6) in patients ≥65 years who received an AIV. The median age was significantly higher in those ≥65 years who received an AIV than those who received an NAIV (Mann–Whitney, *p* < 0.05); also, the median age was significantly higher in both elderly groups than in adults (Mann–Whitney, *p* < 0.05). Sex was only recorded from season 2006–2007, so only 56.0% (*n* = 2150) of the patients presented sex data. From patients of 15–64 years old who received an NAIV, 61.4% (*n* = 499) had sex data and men represented 47.7%; from patients ≥65 years who received an NAIV, data were collected only in 28.1% (*n* = 440) of them, and men represented 50.5%; in turn, from patients ≥65 years who received an AIV, 94.8% (*n* = 1148) had sex data collected, and men represented 44.7% of the patients. A total of 477 serum samples were analyzed for classical A(H1N1), 372 for A(H1N1) pdm09 and 669 for A(H3N2) in adults; 1366, 227 and 1412 serum samples for classical A(H1N1), A(H1N1)pdm09 and A(H3N2), respectively, in elderly receiving an NAIV; and 428, 909 and 874 serum samples for classical A(H1N1), A(H1N1) pdm09 and A(H3N2), respectively, in elderly receiving an AIV.

### 3.2. Humoral Protection before vaccination

First, before vaccination, a descriptive analysis and a comparison of the humoral status of each cohort for every influenza subtype, in terms of GMTs and SPR, was performed (Table 1).

In adults (15–64 years), GMTs against the classical A(H1N1) subtype (53.0, CI 95%: 45.5–60.6) were significantly lower than A(H1N1)pdm09 (85.6, CI 95%: 69.4–96.3) and A(H3N2) (81.0, CI 95%: 71.4–91.3), but no differences were found between these last two subtypes (ANOVA, Kruskal–Wallis *p* < 0.05). In the elderly vaccinated with an NAIV, no significant differences were found between classical A(H1N1) (37.1, CI 95%: 34.7–39.8) and A(H1N1)pdm09 (37.9, CI 95%: 32.0–46.6), but both presented significantly lower GMTs than the A(H3N2) subtype (85.7, CI 95%: 79.7–92.6). Finally, the elderly receiving an AIV showed significantly different pre-vaccination GMTs between all subtypes; the lowest GMTs were observed in the classical A(H1N1) (23.56, CI 95%: 21.3–26.2) subtype, followed by the A(H1N1)pdm09 subtype (56.2, CI 95%:47.6–57.6) and then A(H3N2) (79.7 CI 95%: 71.7–89.2).

Analyzing the pre-vaccination SPR, the adults presented a significantly higher pre-vaccination SPR (Ab titers ≥ 1/40) for the A(H1N1)pdm09 (73.7%) and A(H3N2) (76.2%) subtypes compared to classical A(H1N1) (62.9%), but no significant differences were found among them (Mann–Whitney, *p* < 0.05). In the elderly cohort vaccinated with NAIV, no differences between both the classical and pandemic A(H1N1) subtypes (55.2% and 56.0%, respectively) were found, but both of them presented a significantly lower SPR compared to the A(H3N2) subtype (77.9%). In the case of the elderly cohort vaccinated with AIV, the pre-vaccination SPR was significantly higher for the A(H3N2) (75.7%) than A(H1N1)pdm09 (67.8%) and classical A(H1N1) (40.0%) subtypes. 

**Figure 1:**
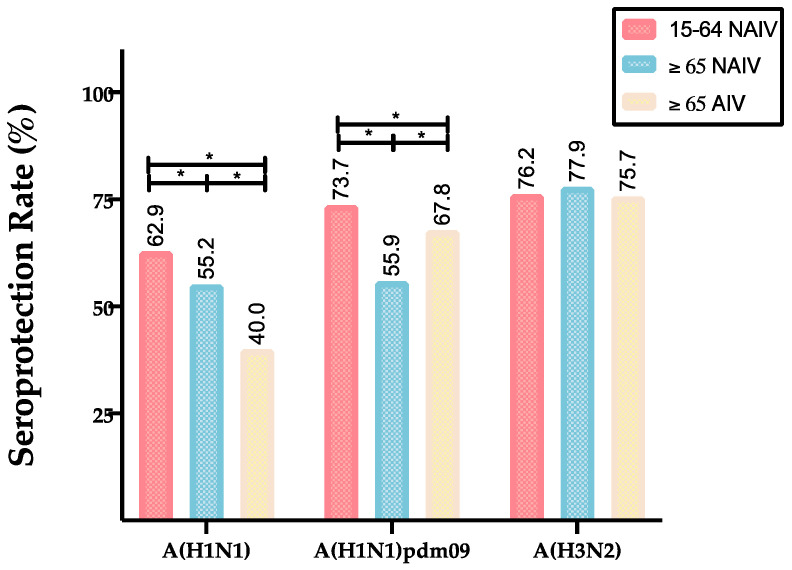
Pre-vaccination SPR of each cohort against each strain/subtype of influenza A. The line marked with * represent statistically significant (*p* < 0.05) differences between those two cohorts.

On the other hand, we compared the pre-vaccine GMTs and SPR for all subtypes between the three different cohorts. Pre-vaccination GMTs showed their highest values in adults for both the classical A(H1N1) (53.0, CI 95%: 45.5–60.6) and A(H1N1) pdm09 (85.6, CI 95%: 69.4–96.3) subtypes (Table 1). However, for the A(H3N2) subtype (85.7, CI 95%: 79.7–92.5), the highest values were achieved in the NAIV elderly cohort (Kruskal–Wallis *p* < 0.05). These differences between pre-vaccine GMTs were also observed when both elderly groups were compared regarding both classical A(H1N1) and A(H1N1) pdm09, but not for A(H3N2). In the case of the A(H1N1)pmd09 subtype, the elderly vaccinated with an AIV showed significantly higher GMTs (56.2 CI 95%: 47.6–57.6) than the other groups, but for the classical A(H1N1) subtype, the elderly vaccinated with an NAIV was the group who showed significantly higher GMTs (37.1, CI 95%: 34.4–39.8). Adults were the group that showed a significantly higher pre-vaccine SPR against classical A(H1N1) (62.9%) and A(H1N1)pdm09 (96.0%), compared to both elderly groups; while, the SPR against the A(H3N2) subtype was similar in those three groups, ranging from 75.7 to 77.9%, and no differences were found among them (Pearson Chi-square, *p* < 0.05) (Figure 1).

### 3.3. Humoral Response after Influenza Seasonal vaccination

The study of the humoral response against seasonal influenza vaccine was based on two different parameters: the analysis of the GMTs and their increase, and the SCR (Table 2).

**Figure 2:**
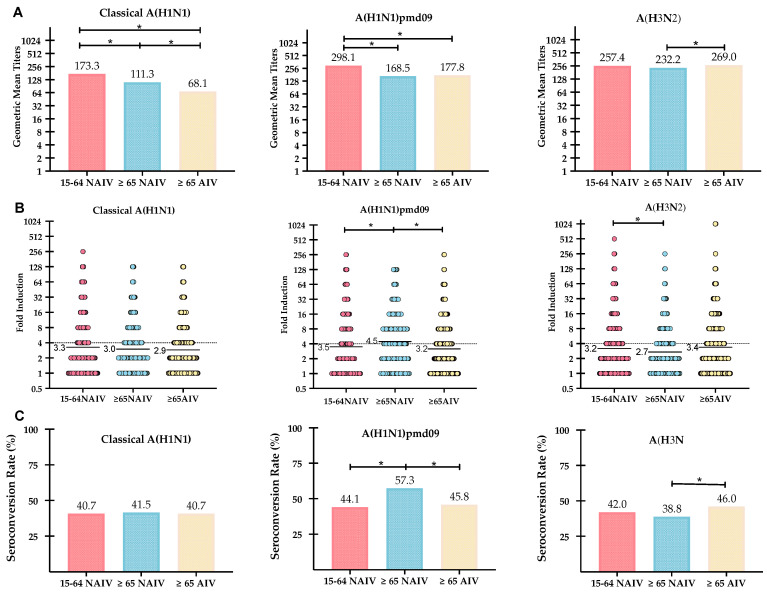
Plots analyzing the effect of the vaccine on the three different age groups for all of the influenza A subtypes analyzed. (**A**) Post vaccination GMT values. (**B**) Individual fold-induction (post/pre-HI titers) marked in dots, and the GMT increase marked with a line. (**C**) Seroconversion rate (percentage of population with 4-folded initial HI titers). Statistical signification between cohorts (*p* < 0.05) is marked with a line and * in all plots.

The GMTs reached after vaccination in adults (15–64 years old) vaccinated with an NAIV were significantly higher against the A(H1N1)pdm09 (298.1, CI 95%: 264.6–333.1) and classical A(H1N1) subtypes (173.3, CI 95%: 154.1–194.4) (Kruskal–Wallis ANOVA; *p* < 0.05) compared to both elderly groups. The elderly vaccinated with an AIV was the cohort that achieved the highest post-vaccination GMTs against the A(H3N2) subtype (269.0, CI 95%: 243.8–297.1), but no significant difference was found between all groups. When both the elderly cohorts were compared, significantly higher GMTs were found for the classical A(H1N1) subtype in the cohort vaccinated with an NAIV (111.3, CI 95%: 103.9–119.3); also, for the A(H3N2) subtype, it was found that the elderly vaccinated with an AIV achieved significantly higher post-vaccination GMTs (269.0 CI 95%: 243.8–297.1) than those vaccinated with the NAIV (Kruskal–Wallis ANOVA; *p* < 0.05) (Figure 2).

The GMT increase induced by the vaccination was significantly higher in the elderly vaccinated with the NAIV for the A(H1N1)pdm09 subtype (4.5) than in adults (3.5), and no other differences were found when comparing both elderly cohorts to the control group of adults (Kruskal–Wallis ANOVA; *p* < 0.05). On the other hand, the GMT increase was significantly higher against the A(H3N2) subtype for the elderly vaccinated with an AIV (3.4) than those vaccinated with the NAIV (2.7), but no differences were observed when the AIV group was compared with the adults. No differences were found regarding the classical A(H1N1) subtype between the three groups analyzed (Kruskal–Wallis ANOVA; *p* < 0.05) (Figure 2). 

When the SCR was analyzed, no significant differences for any subtype in the adult cohort was observed compared to the elderly vaccinated with the AIV. In the case of the NAIV in the elderly cohort, there was a significantly higher SCR against A(H1N1)pdm09 (57.3%) than the elderly vaccinated with the AIV (45.8%) and adults vaccinated with the NAIV (44.1%) (Pearson Chi-square, *p* < 0.05). On the other hand, in the case of subtype A(H3N2), the SCR was significantly higher in those elderly vaccinated with the AIV (46.0%) than those vaccinated with the NAIV (38.8%), and no differences were found between the elderly AIV group and adults (Pearson Chi-square, *p* < 0.05) (Figure 2). 

**Figure 3:**
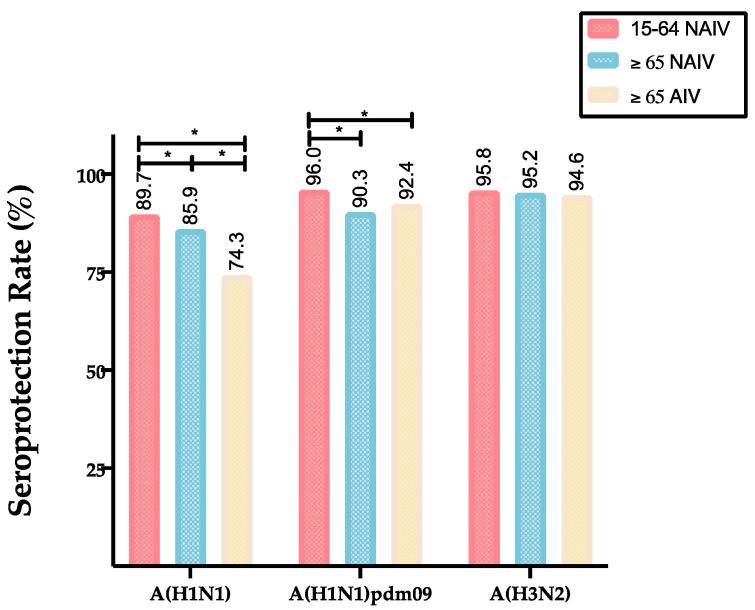
Seroprotection rate after vaccination of different age cohorts against influenza A strains/subtypes. Statistical signification between cohorts (*p* < 0.05) is marked with a line and * in all plots.

The post-vaccine SPR (Abs ≥ 1/40) in the adult group showed the highest values against all the viruses analyzed (A(H1N1)pdm09 = 96.0%; classical A(H1N1) = 89.7%); A(H3N2) = 95.8%) compared to the elderly groups (Figure 3). The SPR acquired after vaccination was higher than 90% against A(H1N1)pdm09 and A(H3N2) in all groups, but ranged between 74.3% and 89.7% against the classical A(H1N1) subtype. When comparing the SPR of both the elderly cohorts and the adult cohort, this SPR was significantly higher in the adults for the A(H1N1)pmd09 (96.0%) than the elderly vaccinated with the NAIV (90.3%) and AIV (92.4%), and also against the classical A(H1N1) subtype (adults 89.7%; elderly vaccinated with the NAIV 85.9%; elderly vaccinate with the AIV 74.3%). No significant differences were found for the A(H3N2) subtype comparing all groups (Pearson Chi-square, *p* < 0.05). The elderly cohort vaccinated with the NAIV showed a significantly higher post-vaccine SPR against the classical A(H1N1) subtype (85.9%) compared to the elderly vaccinated with the AIV (74.3%) (Pearson Chi-square, *p* < 0.05).

## 4. Discussion

Our data showed that seroprotection prior to influenza vaccination, which was analyzed by calculation of GMTs and SPR, was similar against the A(H3N2) subtype in both elderly groups and adults. However, this seroprotection was significantly lower in both elderly groups compared to adults for both A(H1N1)pmd09 and the classical A(H1N1). Our results showed that age is related with the declining of the humoral protection against both A(H1) subtypes (classical and pandemic), but not in the case of A(H3N2). 

Some studies suggest that infection with A(H1N1) or A(H3N2) in humans induces different antibody profiles. Infection with A(H1N1) viruses elicit broad antibody responses, whereas infection with the A(H3N2) virus leads to a narrow antibody response [14,15]. In our data, we observed that the A(H1N1) subtypes presented lower pre-vaccination SPR and GMTs than A(H3N2) in the elderly, but also that these values were similar in both elderly groups and adults for the A(H3N2) subtype.

One plausible explanation for the steady GMTs and SPRs, regardless of age, against the A(H3N2) subtype, could be that antibodies created against this subtype, after repeated exposure to vaccination (it has to be remembered that our cohorts have been vaccinated repeatedly, even if exactly which years is not known), were progressively modified, switching their affinity and variability against this virus. Namely, the avidity towards the subtype that has initially imprinted the subject declines as their ability to recognize other antigenic sites of A(H3N2) by B cells arises [16]. According to a study conducted in naïve ferrets [16], repeated vaccination with A(H3) resulted in improved quality of the antibodies, without necessarily increasing the binding affinity, but with an enhanced multivalent binding capacity for diverse antigenic sites, which would explain a sustained protection through the years. 

On the other hand, we have not found any scientific evidence that shows that antibodies against the A(H1N1) subtypes (either classical or pandemic) experience the same behavior; so, according to our data, fading caused by immunosenescence would explain the decrease in protection in the elderly compared to adults, specifically for the A(H1N1) subtypes. The term immunosenescence accounts for the effect of age over immunity, implying alterations in the distribution and cells engaged in innate and adaptative immunity as well as its functions. The ability of an individual to generate an antigenic humoral response for the first time increases rapidly after birth until sexual maturity, just to start declining gradually. This decline is associated with a reduction in antibodies and their avidity, and also entails a decreased T cell help that limits B and T cell responses to pathogens, contributing to an increased susceptibility to infections in the elderly, as well as a decreased response to vaccination in this population [17,18].

Seroprotection acquired after vaccination is not only dependent on the effect of the vaccine, but also on the serological status and previous protection of the population studied. For example, a group of individuals with high pre-vaccine titers hampers a wide serological response, but also high pre-vaccine seroprotection rates would not allow a substantial increment of them either. Our data showed that, after vaccination, the Ab titers achieved were similar against A(H3N2) for adults and elderly vaccinated with AIV, but significantly lower for the elderly vaccinated with NAIV compared to the latter. Nevertheless, significant differences were found in the GMTs achieved against the A(H1N1) subtypes within all three cohorts, being significantly higher in adults in both the classical and pandemic A(H1N1) strains. In addition, the SPRs achieved after vaccination reached at least to 70% in all cohorts for all A subtypes.

Concerning the parameters that provide information about the vaccine’s effect—that is, the GMT increase and SCR—it is apparent that our data showed a much better performance of the AIV than the NAIV against A(H3N2) in the elderly, even pulling off a higher SCR than adults vaccinated with an NAIV. It was remarkable that the NAIV in the elderly induced a significantly higher response against the pandemic A(H1N1)pdm09 subtype compared to adults vaccinated with the NAIV and the elderly with the AIV. These results are in agreement with a study that suggested that the benefit of influenza vaccination in elderly people might differ depending on the influenza subtypes, and recorded the A(H3N2) subtype to present the highest vaccine efficacy when using an AIV in the elderly compared to the NAIV [19,20]. Therefore, we are facing a dilemma of which vaccine should we use to protect the elderly because, according to our data, each one of the two vaccine types tested seems to stimulate a higher response against one of the subtypes but not against the other. Different studies in many countries have associated higher influenza-associated respiratory mortality in the elderly in seasons dominated by the A(H3N2) subtypes, but largest in adults in seasons dominated by A(H1N1)pdm09. It is also unknown whether the increased burden of A(H3N2) in the elderly is due to higher attack rates, greater clinical severity, or both [21,22,23,24]. 

As previously suggested in other studies, an AIV might be more suitable for the elderly [19]. Despite presenting a significantly lower response for A(H1N1)pdm09, the SCR provided by the AIV vaccine was similar for both subtypes, suggesting that the increase in protection against influenza A(H3N2) provided by the AIV might be clinically important, given the risk that this subtype poses to elderly people; thus, a combination of pre-vaccination immunity and post-vaccination boosting might be sufficient to minimize the risk of infection with the A(H1N1)pdm09 subtype [19]. 

Our data of SPR and GMT increase suggest that, somehow, a significantly improved response is achieved by the elderly vaccinated with the NAIV compared to adults and elderly vaccinated with the AIV, in particular against the A(H1N1)pdm09 subtype. This might suggest the existence of an original antigenic sin phenomenon. The term original antigenic sin (OAS), nowadays known as antigenic seniority, was first described in the 1950s by Thomas Francis, Jr., and coined by himself years later [25,26,27]. The hypothesis suggested that a person’s antibody response is determined by the first influenza infection of their childhood. Upon exposure to new drifted or shifted strains, the immune system recognizes the conserved epitopes and back-boosts memory cells from the first infections, generating antibodies that recognize common epitopes rather than create de novo protective responses from naïve B cell populations [28]. In our study, in both of the elderly cohorts the youngest person would have been born before 1953, so they would have been first imprinted with the classical A(H1N1) strains that circulated in the first half of the 20th century, which, as some studies have shown, share at least a 70% genetic and antigenic similarity with the A(H1N1)pdm09 subtype [7,29,30]. However, this phenomenon has not been observed in the group vaccinated with the AIV, which presents a comparable although significantly higher median age than the NAIV group (81 years versus 75 years); so, as far as we are concerned, we ignored any singularity that stimulates the occurrence of this phenomenon. Be that as it may, our data suggest that the NAIV could have triggered an OAS for the A(H1N1)pdm09 subtype.

One of the weakness of our study is that the data had been collected over a long period of time, accounting for 21 influenza seasons, which entails a wide dispersion of data in terms of age that might result in bias. In addition, the AIV was introduced in 2006, so there is no data prior to that season. On the other hand, the fact of having such a wide-ranging study provides us with a global picture of the humoral response to influenza vaccination. 

Another limitation of the study is that antibody response was assessed by the hemagglutination inhibition (HI) assay that, to date, is considered the “gold standard” test for the evaluation of vaccine-induced antibody responses by the WHO [10], as it only evaluates antibody responses against the head of the haemagglutinin; this could result in information forfeited in terms of antibody responses against other antigenic proteins of the virus, such as the stalk domains.

## 5. Conclusions

In summary, our results show that the AIV induce a higher antibody response against the A(H3N2) subtype in the elderly than the NAIV, while the latter shows a higher response against the A(H1N1)pm09 subtype. Despite the differences shown by the distinct vaccine types employed against the different influenza A subtypes, in all cases the SCR achieved were over 40%, reaching a vaccine SPR of at least 74.3% against any of the subtypes, and an antibody GMT increase above 2.71. Our data prove that vaccination in a population ≥ 65 years old increases in any case the antibody titers against any of the influenza A subtypes and, therefore, supports the annual vaccine recommendation for this age group. With regard to the higher mortality in the elderly in the case of A(H3N2) epidemics, the use of an AIV would appear as more advisable for that age rank.

## Figures and Tables

**Table 1:** Protection presented by each cohort before vaccination in terms of the geometric mean titers (GMTs) and seroprotection rate (SPR) against each strain/subtype of influenza A. Statistical significance within each cohort, comparing all influenza A subtypes against each other. * *p*-values < 0.05.

Vaccinated Cohorts	Values	Strain/Subtype			Statistical Significance (*p*-Value)		
	Pre vaccine	A(H1N1)	A(H1N1) pdm09	A(H3N2)	A(H1N1) vs. A(H1N1) pdm09	A(H1N1) vs. A(H3N2)	A(H1N1) pdm09 vs. A(H3N2)
15–64 NAIV	GMTs (CI 95%)	53.0 (45.5–60.6)	85.6 (69.4–96.3)	81.0 (71.4–91.3)	0.000 *	0.000 *	1.000
	SPR (%)	62.9	73.7	76.2	0.001 *	0.000 *	0.365
≥65 NAIV	GMTs (CI 95%)	37. 1(34.7–39.8)	37.9 (32.0–46.6)	85.7 (79.7–92.5)	1.000	0.000 *	0.000 *
	SPR (%)	55.2	56.0	77.9	0.833	0.000 *	0.000 *
≥65 AIV	GMTs (CI 95%)	23.6 (21.3–26.2)	56.2 (47.6–57.6)	79.7 (71.7–89.2)	0.000 *	0.000 *	0.000 *
	SPR (%)	40.0	67.8	75.7	0.000 *	0.000 *	0.000 *

**Table 2:** Post-vaccination status and effect of each cohort against each influenza A strain/subtype in terms of the GMTs (geometric mean titers), SPR (seroprotection rate), GMT increase (post-GMTs/pre-GMTs) and SCR (4-fold induction).

Influenza Viruses	Values	Vaccinated Cohorts		
Strain/Subtype	Post-vaccination	≥65 NAIV	≥65 AIV	15–64 NAIV
A(H1N1pdm09)	GMTs (CI 95%)	168.5 (143.5–198.2)	177.8 (155.7–183.7)	298.1 (264.6–333.1)
	SPR (%)	90.3	92.4	96.0
	GMTs increase	4.5	3.2	3.5
	SCR (%)	57.3	45.8	44.1
A(H1N1)	GMTs (CI 95%)	111.3 (103.9–119.3)	68.2 (61.2–76.1)	173.3 (154.1–194.4)
	SPR (%)	85.9	74.3	89.7
	GMTs increase	3.0	2.9	3.3
	SCR (%)	41.5	40.7	40.7
A(H3N2)	GMTs (CI 95%)	232.2 (217.7–247.0)	269.0 (243.8–297.1)	257.4 (230.7–285.5)
	SPR (%)	95.2	94.6	95.8
	GMTs increase	2.7	3.4	3.2
	SCR (%)	38.8	46.0	42.0
